# Chemigenetic Ca^2+^ indicators report elevated Ca^2+^ levels in endothelial Weibel-Palade bodies

**DOI:** 10.1371/journal.pone.0316854

**Published:** 2025-01-27

**Authors:** Julian Terglane, Nicole Mertes, Sarah Weischer, Thomas Zobel, Kai Johnsson, Volker Gerke

**Affiliations:** 1 Institute of Medical Biochemistry, Center for Molecular Biology of Inflammation, University of Muenster, Muenster, Germany; 2 Department of Chemical Biology, Max Planck Institute for Medical Research, Heidelberg, Germany; 3 Münster Imaging Network, Cells in Motion Interfaculty Centre, University of Muenster, Muenster, Germany; Institut Curie, FRANCE

## Abstract

Weibel-Palade bodies (WPB) are secretory organelles exclusively found in endothelial cells and among other cargo proteins, contain the hemostatic von-Willebrand factor (VWF). Stimulation of endothelial cells results in exocytosis of WPB and release of their cargo into the vascular lumen, where VWF unfurls into long strings of up to 1000 µm and recruits platelets to sites of vascular injury, thereby mediating a crucial step in the hemostatic response. The function of VWF is strongly correlated to its structure; in order to fulfill its task in the vascular lumen, VWF has to undergo a complex packing/processing after translation into the ER. ER, Golgi and WPB themselves provide a unique milieu for the maturation of VWF, which at the level of the Golgi consists of a low pH and elevated Ca^2+^ concentrations. WPB are also characterized by low luminal pH, but their Ca^2+^ content has not been addressed so far. Here, we employed a chemigenetic approach to circumvent the problems of Ca^2+^ imaging in an acidic environment and show that WPB indeed also harbor elevated Ca^2+^ concentrations. We also show that depletion of the Golgi resident Ca^2+^ pump ATP2C1 resulted in only a minor decrease of luminal Ca^2+^ in WPB suggesting additional mechanisms for Ca^2+^ uptake into the organelle.

## Introduction

Weibel-Palade bodies (WPB) are elongated secretory organelles characteristic for endothelial cells that harbor an assortment of different cargo proteins [1–14]. Stimulation of endothelial cells with Ca^2+^ and cAMP rising agonists triggers exocytosis of WPB and secretion of its stored cargo into the vascular lumen thereby enabling the cells to participate in a variety of physiological processes including hemostasis, inflammation and angiogenesis [15].

The main cargo of WPB is the multidomain protein von Willebrand factor (VWF) which serves important functions in preventing excessive bleeding after blood vessel injury. VWF itself drives the formation of WPB at the trans-Golgi network (TGN) and recruits additional cargo proteins to the organelle [3, 16–25]. The physiologically relevant form of VWF is the multimer; VWF multimers form long strings of concatenated polypeptide chains that are highly potent in recruiting platelets to the site of vascular injury thereby initiating the formation of a platelet plug that stops bleeding [26–29]. Hence, upon traversing the secretory pathway VWF has to undergo a complex conversion in order to be stored in a multimeric and ready-to-be-released form. For this process the ER, Golgi and the storage organelle, WPB, provide a unique environment.

VWF is co-translationally synthesized as a preproprotein into the ER where it subsequently undergoes a C-terminal dimerization by disulfide bridging [30]. VWF continues to dimerize in the Golgi stacks via noncovalent interactions and forms a structure described in the literature as dimeric bouquet [31, 32]. In the TGN, VWF dimers assemble into tubules and multimerize at the N-terminus via formation of disulfide bonds between D´D3 domains of proVWF dimers in neighbouring positions [32–35]. At this stage, the propeptide is cleaved by the Ca^2+^-dependent protease furin; however, it remains noncovalently associated to the tubulated mature protein [35–38]. In a final step dictated by the organization of the Golgi stacks, multiple VWF quanta are co-packed into newly budding WPB [31]. Subsequently, VWF tubules become tightly packed as the organelle matures and is transported along the microtubule network to the cell periphery [34, 39]. Here, mature WPB are anchored at the actin cytoskeleton awaiting a stimulus for secretion [39–41].

Tubulation, multimerization and tight packing of VWF tubules are crucial for the formation of proper WPB and a prequesite for the hemostatic protein to fulfill its function [24, 26, 28, 29, 34]. Correct packing of VWF in WPB strongly depends on a low organelle pH that requires activity of a WPB-associated V-ATPase [13, 28, 32, 33, 35, 42–47]. Apart from the well-documented presence of elevated proton concentrations in the lumen of WPB [48, 49], nothing is known about ion homeostasis inside WPB. However, such data are important as in vitro analyses indicate that the presence of elevated Ca^2+^ levels may be required for tubulation and binding of the propeptide to mature VWF [33, 35, 50]. Enrichment of protons and Ca^2+^ and even other ions is a key feature of many secretory organelles and LROs such as insulin granules and melanosomes, and this specific ionic milieu is required for proper processing and storage of cargo in these organelles [51–54]. In the case of Ca^2+^, it has also been shown that some of the secretory organelles/LROs are equipped with an appropriate machinery for Ca^2+^ uptake and release which enables them to participate in Ca^2+^ signaling [55–61].

Although Ca^2+^ is required for proper VWF packing/processing in vitro, it is not known whether WPB indeed contain elevated Ca^2+^ concentrations to fulfill this purpose in cells. To address this, we exploited the ability of the self-labeling HaloTag to be genetically targetable to a subcellular compartment and to bind covalently to synthetic dyes, which are taken up by living cells and show an altered fluorescence in response to Ca^2+^ binding [62, 63]. Targeting Ca^2+^ dye-binding HaloTag constructs to WPB revealed that these secretory organelles contain elevated Ca^2+^ levels most likely required for proper VWF maturation. Furthermore, we show that excessive loading of WPB with Ca^2+^-chelating dyes and depletion of the Ca^2+^-transporting ATPase ATP2C1, a Golgi resident protein also found in the WPB proximity proteome, results in the formation of slightly smaller WPB and in a minor decrease of luminal Ca^2+^ in WPB.

## Results

### Visualization of intraluminal Ca^2+^ in WPB

A critical feature of many secretory organelles and LROs such as lysosomes, melanosomes, synaptic vesicles and insulin granules is a low pH often accompanied by an intraluminal accumulation of Ca^2+^ [52–54, 64, 65]. Furthermore, work employing purified D1D2 and D´D3 domains of VWF suggests that the presence of Ca^2+^ is required for tubulation of the protein and it was shown that the noncovalent interaction of cleaved D1D2 propeptide homodimers with VWF multimers inside WPB depends on Ca^2+^ [33, 35]. Therefore, we aimed to determine whether WPB also harbor elevated concentrations of Ca^2+^, which could be required for proper VWF tubulation and packing. We first addressed this experimentally in live cells by targeting the genetically encoded Ca^2+^ indicator GCaMP6s to the organelle. GCaMP constitutes a circularly permuted EGFP fused to calmodulin (CaM) and the M13 fragment of myosin light chain kinase with EGFP protonated and only weakly fluorescent in the absence of Ca^2+^. Upon Ca^2+^ binding, CaM tightly binds M13 leading to conformational changes that shield EGFP from surrounding water and result in greatly increased fluorescence, thus permitting the use as Ca^2+^ indicator [66, 67]. The targeting of GCaMP6s to the lumen of WPB was achieved by fusing it in tandem to mApple and the luminal domain of P-selectin, a bona-fide WPB cargo herein referred to as P-sel-lum (S1 Fig in S1 File). Following ectopic expression in primary human endothelial cells (HUVEC) the construct was almost exclusively present in WPB as revealed by the mApple fluorescence. However, no GCaMP6s signal could be detected inside the organelle. As the fluorescence of GCaMPs is pH sensitive and WPB are characterized by a luminal pH of appr. 5.5 we analyzed whether the lack of GCaMP6s signal could be due to the rather acidic environment inside WPB. Therefore, we neutralized the low internal pH of WPB by treating cells with bafilomycin A (BafA1), an inhibitor of the V-ATPase proton pump which is also present on WPB and responsible for luminal acidification [44]. Proton pump inhibition resulted in clear GCaMP6s fluorescence signals in construct-bearing (i.e. mApple-positive) WPB, demonstrating the sensitivity of GFP-derived indicators for an acidic environment [68–71].

**Fig 1:**
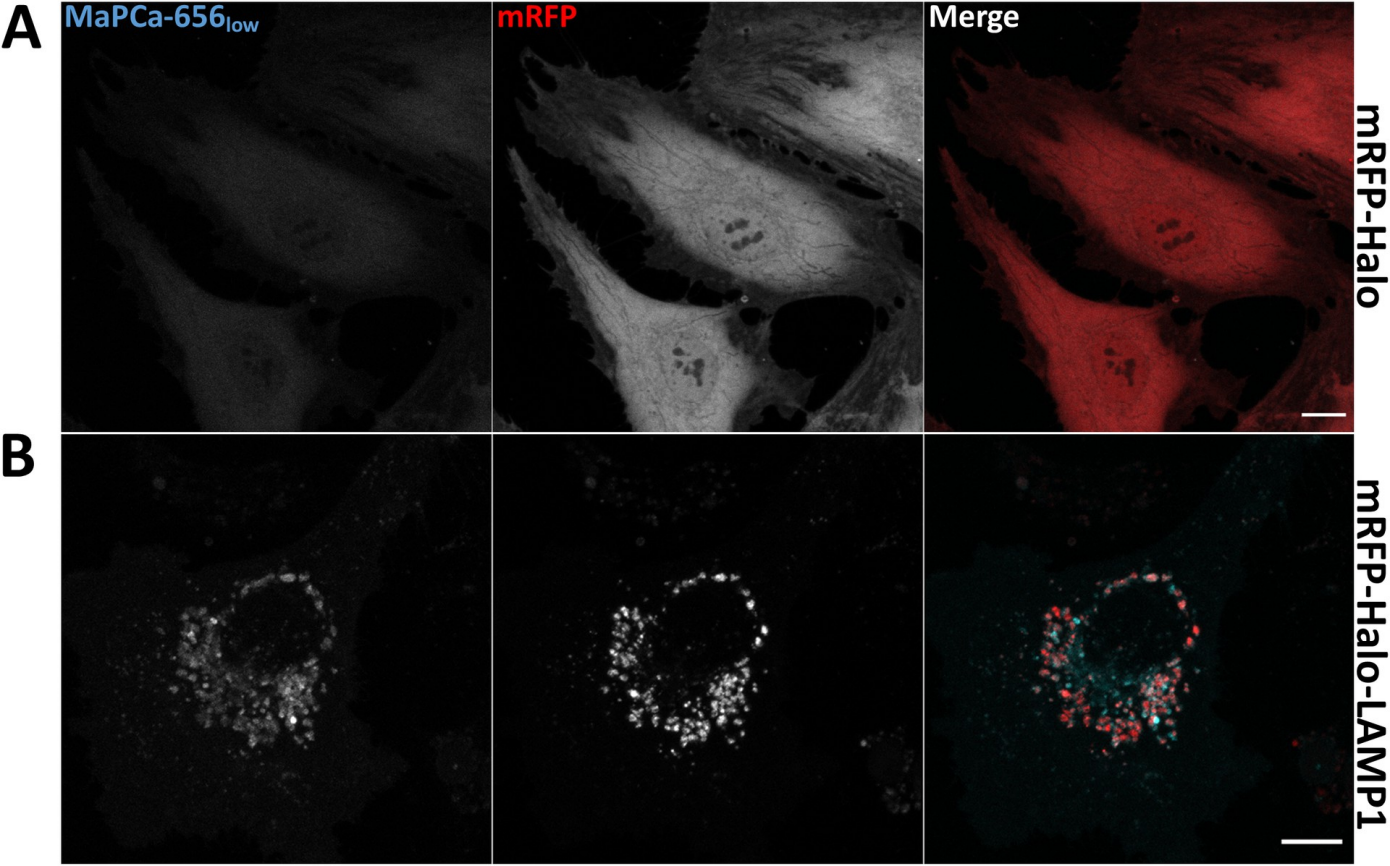
MaPCa-656_low_ reports elevated Ca^2+^ concentrations in lysosomes. HUVEC transfected with either mRFP-Halo (**A**) or mRFP-Halo-LAMP1 (**B**) were incubated with 1 µM MaPCa-656_low_ for 2 h at 37 °C and subjected to live cell microscopy. Note that not all mRFP-Halo-LAMP1 positive lysosomes show enhanced MaPCa-656_low_ fluorescence. Shown are confocal microscopy images displaying maximum intensity projections of z-stacks. Note the cytosolic (A) or lysosomal recruitment (B) of the Ca^2+^ indicator MaPCa-656_low_. Scale bars: 10 μm.

**Fig 2:**
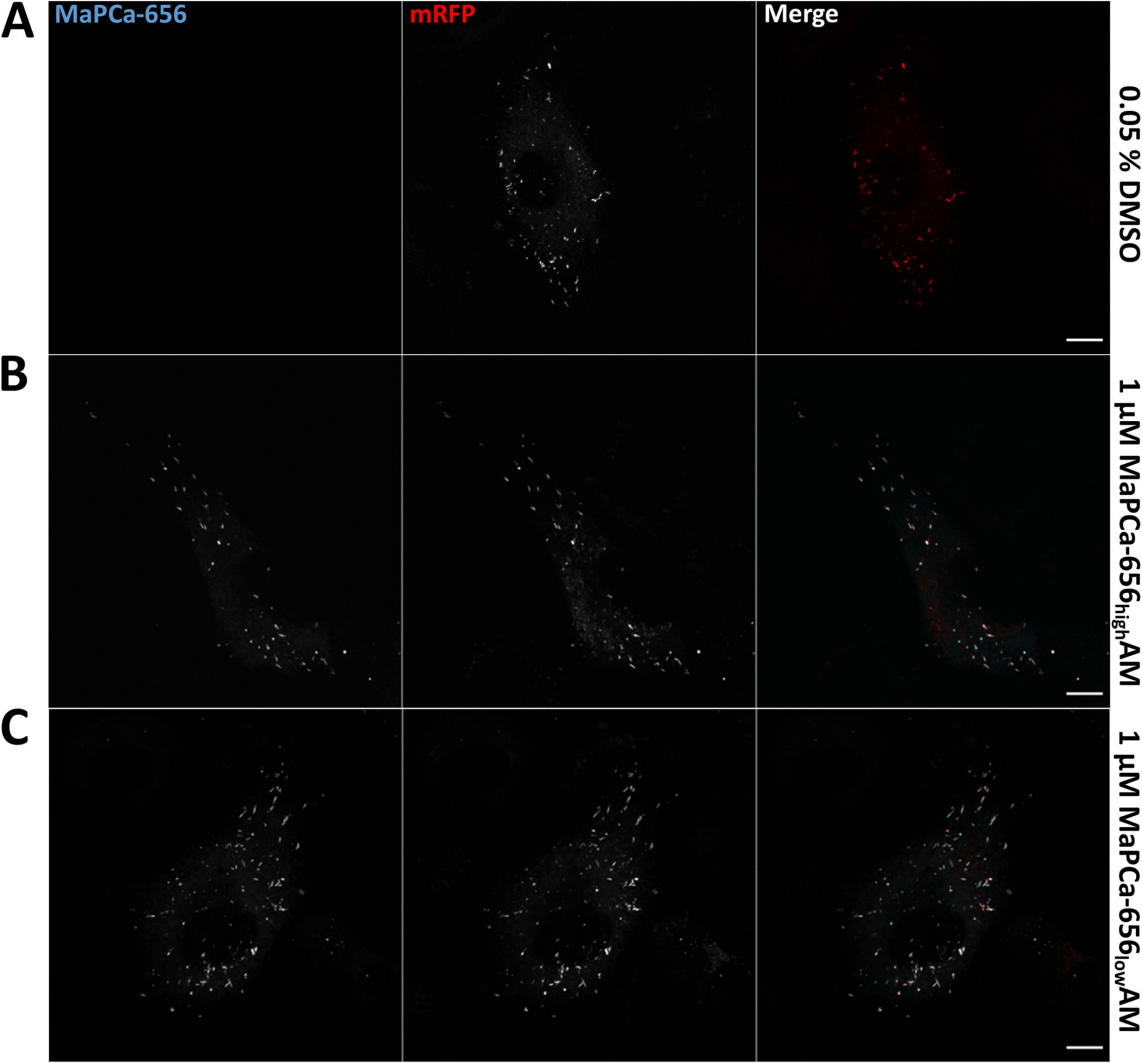
MaPCa-656 detects Ca^2+^ in the acidic milieu of VWF-mRFP-Halo positive WPB. 24 h after transfection with a plasmid encoding VWF-mRFP-Halo, confluent HUVEC were incubated with 0.05% DMSO (**A**), 1 µM of the high affinity Ca^2+^ indicator MaPCa-656_high_ (**B**) or 1 µM of the low affinity Ca^2+^ indicator MaPCa-656_low_ (**C**) for 2 h at 37 °C and subsequently subjected to live cell imaging. Note that expression of VWF-mRFP-Halo recruits the Ca^2+^ indicators successfully to WPB and reports elevated Ca^2+^ concentrations in the organelle. In the entire HUVEC population all WPB positive for the VWF-mRFP-Halo construct (i.e. positive for mRFP fluorescence) also show enhanced MaPCa-656_low_ and MaPCa-656_high_ fluorescence. Shown are confocal microscopy images displaying maximum intensity projections of z-stacks. Scale bars: 10 μm.

To overcome this inherent limitation of fluorescent protein-based indicators [68, 72, 73] which in the case of WPB, led to the failure to monitor elevated Ca^2+^ levels at the low luminal pH of the unperturbed organelle via GCaMP6s fluorescence, we aimed to combine a genetic targeting strategy with the lower pH sensitivity, higher brightness and photostability of synthetic Ca^2+^ indicators [74–77]. Therefore, an organelle targeting protein was fused in tandem to the less pH-sensitive fluorescent protein mRFP1 (pKa = 4.5) [68, 78] and to the self-labeling HaloTag, which enables the binding of synthetic indicator moieties [62]. For this chemigenetic approach we utilized the Ca^2+^ indicator MaPCa-656 developed and characterized earlier [63]. MaPCa-656 comprises a si-rhodamine dye equipped for Ca^2+^ binding with BAPTA (high-affinity chelator, MaPCa-656_high_) or the MOBHA (low-affinity chelator, MaPCa-656_low_) moieties and attached to a chloralkane linker in order to enable capturing by the HaloTag. We first verified the binding of MaPCa-656 to the Halo-tag in an acidic and proteolytic environment as well as the tolerance of the synthetic indicator (fluorescence and Ca^2+^ binding) towards a low pH by targeting mRFP-Halo to lysosomes, the classical acidic organelle. This was achieved by fusion with the lysosome-resident protein LAMP1 as shown in immunostainings of transfected HUVEC with anti-LAMP1 and anti-Halo antibodies (S2A Fig in S1 File, Table 1). Incubation with MaPCa-656_low_ for 2 h at 37 °C 24 h post transfection showed a labeling of lysosomal structures and reported, based on the fluorescence signal, an elevated intralysosomal concentration of Ca^2+^ (Fig 1B) compared to the cytosol (Fig 1A). Next, we recruited the MaPCa-656 indicator to WPB by fusing mRFP-Halo to the main cargo of WPB, the glycoprotein VWF. Correct targeting of the construct was verified by the exclusive localization of the mRFP and the anti-HaloTag signal to WPB (S2B Fig in S1 File). HUVEC expressing the VWF-mRFP-Halo construct were then incubated for 2 h at 37 °C with either the MaPCa-656_low_ or the MaPCa-656_high_ Ca^2+^ indicator. Both indicators showed bright signals in VWF-mRFP-Halo-positive WPB revealing that WPB contain elevated Ca^2+^ concentrations (Fig 2B and 2C). No major difference between the signals of MaPCa-656_low_ and MaPCa-656_high_ recruited to WPB could be observed, suggesting that the intraluminal Ca^2+^ concentration of WPB is sufficient to induce a MaPCa-656_low_ response, i.e. in the micromolar range (K_D_ of MaPCa-656_low_ is 457 μM; [63]). Interestingly, there was no significant correlation of the WPB distance from the nucleus to the intraluminal Ca^2+^ content as estimated by the ratio of the MaPCa-656_low_ to the mRFP signal (S3B Fig in S1 File). As more perinuclear localized WPB are considered less mature as compared to the mature peripheral organelles [15], this finding suggest, that already less mature WPB are characterized by elevated Ca^2+^ levels. It should be noted here that an impairment of this chemigenetic approach by FRET between mRFP1 and the si-rhodamine moiety of the MaPCa-656 indicators within the tightly packed WPB could be excluded because acceptor photo bleaching resulted in a FRET efficiency with an average of -17.3 (S4 Fig in S1 File).

**Table 1:** Antibodies used in this study.

Antibody	Manufacturer	Dilution (Application)
ms-α-VWF (F8/86)	DAKO (M0616)	1:500 (IF)
rb-α-VWF	DAKO (A0082)	1:30.000 (IF)
rb-α-Halo	Promega (G928A)	1:100 (IF)
ms-α-LAMP-1 (H4A3)	DSHB	1:200 (IF)
ms-α-HA (16B12)	BioLegend (901502)	1:100 (IF)
ms-α-GM130	BD Biosciences (610823)	1:500 (IF)
rb-α-ATP2C1	Proteintech (13310-1-AP)	1:20 (IF)
ms-α-ATP2C1	Abnova (H00027032-M01)	1:500 (WB), 1:50 (IF)
rb-α-Tubulin (11H10)	CellSignaling (2125)	1:1000 (WB)
dk-α-rb-Alexa Fluor 488™	Invitrogen (A-21206)	1:400 (IF)
gt-α-ms-Alexa Fluor 647™	Invitrogen (A-21235)	1:400 (IF)
dk-α-rb-Alexa Fluor 594™	Invitrogen (A-21207)	1:400 (IF)
dk-α-ms-Alexa Fluor 488™	Dianova (715-545-150)	1:400 (IF)
dk-α-ms IRDye^®^ 800CW	LICOR Biotech (926–32212)	1:5000 (WB)
dk-α-rb IRDye^®^ 680RD	LICOR Biotech (926–68073)	1:5000 (WB)

### Effect of intra-organelle Ca^2+^ on the morphological appearance of WPB

**Fig 3:**
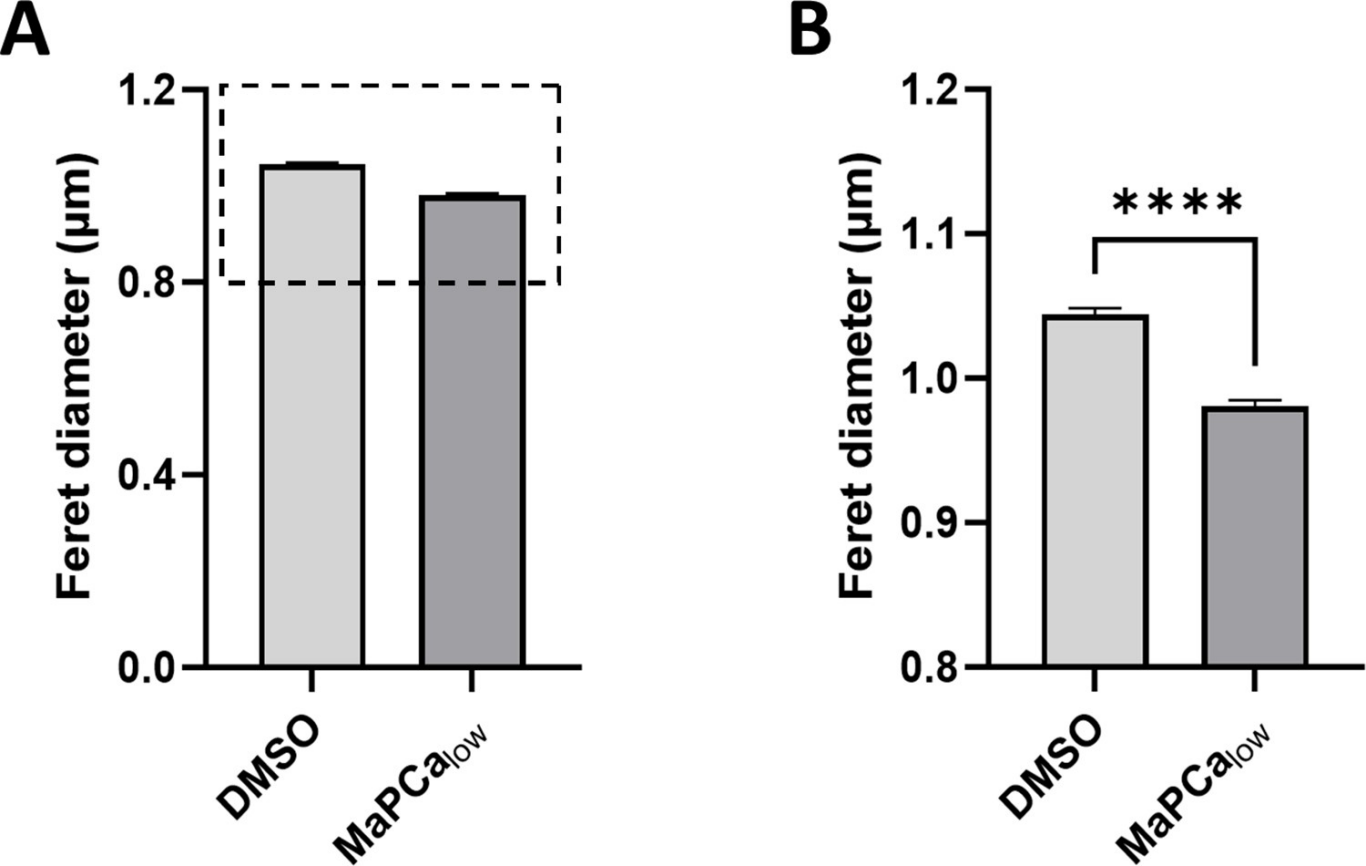
Extensive loading of WPB with the synthetic MaPCa Ca^2+^-Indicators affects WPB size. 4 to 6 h after transfection with a plasmid encoding VWF-mRFP-Halo, HUVEC were incubated with 0.05% DMSO and 1 µM MaPCa-656_low_ for 20 h at 37 °C and subsequently subjected to live cell imaging. The Feret diameter of WPB was determined using the mRFP signal (**A**). In total 4 experiments were conducted (DMSO: 9567 WPB, MaPCa-656_low_: 7834 WPB). Boxed area is shown enlarged on the right side (**B**). Bars indicate the mean. Error bars show SEM. Statistics were conducted using a Mann-Whitney test. ****p≤ 0.0001, ***p≤ 0.001, **p≤0.01, *p≤0.05.

The presence of elevated Ca^2+^ concentrations within WPB provokes questions concerning its origin and function in the context of the organelle. To examine whether Ca^2+^ fulfills a structural role, possibly by affecting the tubulation of VWF or compaction of VWF tubules, we determined the Feret diameter of the organelle, as it can serve as an indicator for the extent of VWF packing [28, 42, 79]. Cells were incubated for 20 h with MaPCa-656_low_ or DMSO as control, because the MaPCa-656 indicators, targeted with high specificity to WPB, are not only able to report Ca^2+^ but can also, in particular upon long term treatment, act as Ca^2+^ chelator reducing the free Ca^2+^ concentration available for binding to the D1D2 and D´D3 domains of VWF. While VWF-mRFP-Halo-positive WPB in cells treated with DMSO exhibit the longest size with an average Feret diameter of 1.04 µm, cells treated with MaPCa-656_low_ are characterized by somewhat smaller WPB with an average Feret diameter of 0.98 µm (Fig 3). This suggest that reduction of the levels of free available Ca^2+^ within WPB results in defects in correct VWF compaction and/or tubulation and as a consequence in somewhat shorter organelles.

**Fig 4:**
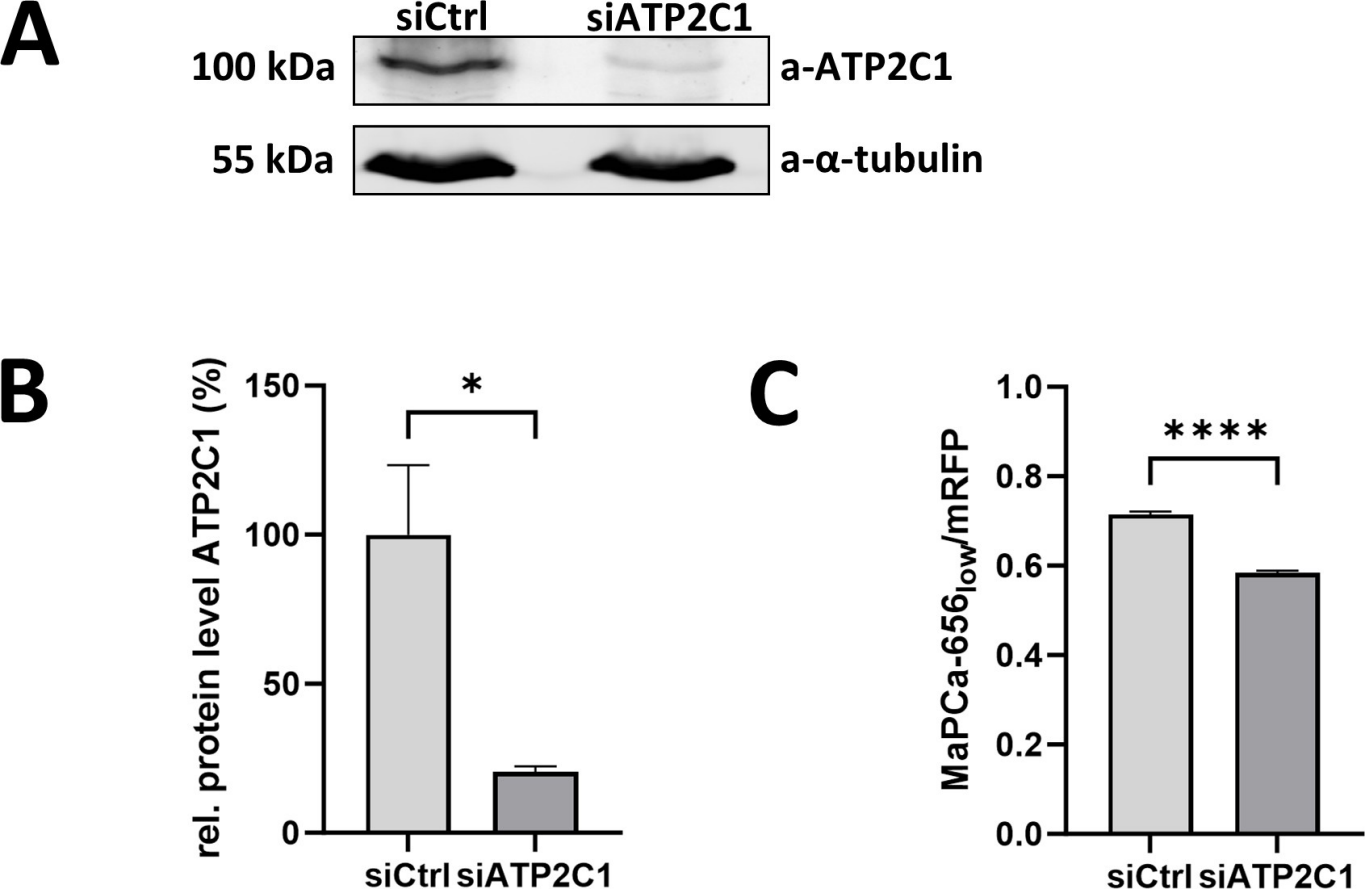
Knockdown of ATP2C1 results in a minor decrease of [Ca^2+^] in WPB. **A.** 24 h post transfection of HUVEC with the respective siRNA cells were lysed and subjected to Western blot analysis with anti-ATP2C1 antibodies. Probing with anti-tubulin antibodies served as loading control. One representative blot is shown on the left (see S8 Fig in S1 File for the entire blot). **B.** ATP2C1 band intensities of 3 blots were measured and normalized to intensities of the loading control. Mean of the normalized intensity of the siControl samples was set to 100%. Bars indicate mean and error bars show standard deviation. Statistics were conducted using an unpaired t-test with Welch´s correction. ****p≤ 0.0001, ***p≤ 0.001, **p≤0.01, *p≤0.05. **C.** Cells transfected with the respective siRNA were additionally transfected with VWF-mRFP-Halo and incubated with 1 µM MaPCa-656_low_ for 2 h at 37 °C. MaPCa-656 and mRFP intensities of single WPB were measured during live cell microscopy in 3 independent experiments (siCtrl: 5943 WPB, siATP2C1: 6285 WPB). Bars indicate the mean. Error bars show SEM. Statistics were conducted using a Mann-Whitney test. ****p≤ 0.0001, ***p≤ 0.001, **p≤0.01, *p≤0.05.

WPB originate at the TGN which maintains free Ca^2+^ in the micromolar concentration range mainly due to activity of the Ca^2+^ ATPase ATP2C1 [80–84]. ATP2C1 was also identified as a WPB-associated candidate protein in a previous APEX2-based proximity proteomic approach employing the WPB-bound GTPase Rab3b as bait [85]. As this suggested that ATP2C1 could be the Ca^2+^ pump responsible for loading WPB with Ca^2+^, we analyzed the intracellular distribution of the enzyme in HUVEC by employing immunofluorescence stainings with a set of commercial anti-ATP2C1 antibodies (see Table 1). No obvious costaining with VWF-positive WPB was observed although the antibodies also do not show a clear Golgi labeling either. Therefore, we also employed a HA-tagged ATP2C1 construct, which was ectopically expressed in HUVEC. However, no obvious WPB targeting of this construct was observed suggesting that if at all only a small fraction of the endothelial ATP2C1 is found on WPB (S5 Fig in S1 File). We next investigated whether ATP2C1 activity could participate in the enrichment of Ca^2+^ inside WPB. Therefore, we depleted the protein via siRNA-mediated knockdown to approximately 20% of the initial level (Fig 4A and 4B) and subsequently analyzed the intra-WPB Ca^2+^ levels using our chemigenetic approach (Fig 4C). Upon knockdown no major changes in the Golgi morphology could be observed as judged by GM130 fluorescence (S6 Fig in S1 File) and as seen in some but not all other studies using a similar approach [82, 83, 86]. However, the knockdown resulted in a very small decrease in the Feret diameter of WPB (S7 Fig in S1 File) as observed in cells expressing VWF-mRFP-Halo and which were loaded with MaPCa-656_low_. Next, we estimated WPB-resident Ca^2+^ levels in these cells by measuring MaPCa intensities in WPB normalized to the expression of VWF-mRFP-Halo. This revealed that Ca^2+^ is slightly but significantly decreased in siATP2C1 as compared to control siRNA treated HUVEC (Fig 4C). Thus, ATP2C1 could at least in part be responsible for generating elevated intraluminal Ca^2+^ concentrations in WPB, possibly at the level at the TGN or even in WPB themselves.

## Discussion

The proper packing of the hemostatic VWF requires a decrease in pH along the secretory pathway as well as elevated Ca^2+^ concentrations in the Golgi and most likely, in the VWF storage organelle, the WPB, itself. While the Golgi is established as a significant intracellular Ca^2+^ store, Ca^2+^ within WPB or a WPB-associated machinery for Ca^2+^ uptake has not been described so far. This study introduces a chemigenetic Ca^2+^ indicator approach that recorded elevated Ca^2+^ concentrations in WPB. Furthermore, targeting of the synthetic Ca^2+^-buffering moiety to WPB resulted in a small decrease in the Feret diameter of the organelle and the Ca^2+^ content within WPB was slightly affected by siRNA-mediated depletion of the Golgi-resident Ca^2+^ pump ATP2C1.

Ca^2+^ was shown to be required for VWF tubulation and association of the propeptide with mature VWF in vitro [33, 35, 50]. However, so far it was not addressed whether WPB indeed contain elevated Ca^2+^ concentrations. Most likely, this lack of information arose from the difficulty of imaging Ca^2+^ in an acidic environment. pH-sensitivity of a Ca^2+^ indicator such as GCaMP6s used in this study can be caused either by the Ca^2+^ sensing unit or by the fluorophore itself. Fluorescent proteins like GFP, which are incorporated in genetically encoded calcium indicators (GECIs) are intrinsically pH sensitive as a low pH quenches their fluorescence for example via an shift in the absorption spectrum (chromophore protonation) and a decrease in quantum yield (thermal relaxation) [68, 72, 73, 87]. The sensitivity in an acidic environment was also observed in our study upon targeting of the GECI GCaMP6s to WPB. Mature WPB maintain a pH of appr. 5.5. [48, 49] and thus, GCaMP6s fluorescence was quenched efficiently. Accordingly, increasing the intraluminal pH by blocking the WPB associated V-ATPase increased GCaMP6s fluorescence and hinted at the presence of Ca^2+^ in the organelle (see S1 Fig in S1 File). Synthetic small-molecule fluorophores were the first fluorescent Ca^2+^ indicators developed [88]. In contrast to GECIs they display superior brightness, higher photostability and are not necessarily intrinsically pH-sensitive but also require synthesis, cellular uptake and lack spatial selectivity [74–77]. By employing a chemigenetic approach in which we successfully targeted the rhodamine-based Ca^2+^ indicator MaPCa-656 to VWF-mRFP-Halo-positive WPB we could overcome pH-sensitivity of GECIs and detect elevated Ca^2+^ concentrations within the VWF storage organelle.

The presence of elevated Ca^2+^ in WPB raises two questions: what is its function within the organelle and where does the Ca^2+^ originate from?

In vitro studies of Huang et al. [33] showed that purified VWF propeptides dimerize and interact with purified disulfide linked dimers of D´D3 domains in order to form helical tubules. These interactions and the tubule formation only occur at a low pH (pH 6.2) in combination with 10 mM Ca^2+^. Tubulation of VWF appears to be independent of multimerization [25, 28]; however, as multimer formation and Ca^2+^-dependent propeptide cleavage were shown to occur in the TGN and precede WPB biogenesis, tubulation also seems to take place in this compartment [35]. This was also suggested by electron microscopy studies [34] and is in line with the idea that VWF tubules are required for pushing the Golgi membrane during WPB formation [34, 89]. Hence, Ca^2+^ required for these processes could be provided in the Golgi lumen. However, a recent study showed that also nontubular VWF is added to forming WPB suggesting that tubulation partly occurs in the lumen of the storage organelle and providing a purpose for the presence of elevated Ca^2+^ levels in WPB [90]. In line with the data of Huang et al. [33], the noncovalent interaction between the VWF propeptide and mature VWF, as shown in vitro with purified proteins and purified WPB, also requires a low pH (pH 6.2, 6.4) in combination with the presence of Ca^2+^ (10mM) [35, 50]. Hence, besides being relevant for WPB biogenesis and maturation, the Ca^2+^- and pH-dependent interaction of mature VWF and the propeptide might also be important for proper VWF storage by not only initiating but also maintaining the tubular structure. As shown for an increase in pH, a depletion of Ca^2+^ from WPB might interfere with tubule assembly/compaction and thereby result in structurally altered organelles [28]. This would be in line with our observation that massive loading of WPB with the Ca^2+^ binding dye MaPCa-656 and thereby buffering the relevant free Ca^2+^ concentration in the organelle leads to somewhat shorter WPB (Fig 3).

Besides a structural role in proper VWF storage, Ca^2+^ could also be sequestered by WPB in order to participate in Ca^2+^ signaling. This would require a machinery for rapid uptake and release as it was already described for other secretory organelles and LROs [55–61]. Goretzko et al. showed that endolysosomal Ca^2+^ release by TPC2, an ion channel also present on melanosomes, affected CD63 loading from endolysosomes onto WPB in HUVEC. However, neither TPC2 or TPC1 could be detected on WPB and TPC2 was shown not to participate in stimulated WPB exocytosis [55, 91].

Elevated Ca^2+^ levels in WPB suggest that the Golgi ionic milieu is simply trapped in the lumen of WPB after budding at the TGN and/or that an uptake mechanism exists. For most Ca^2+^ storage organelles, Ca^2+^ loading is performed by their membrane bound ATP-driven Ca^2+^ pumps, SERCA or ATP2C1 [61]. Also described are ion exchanger systems, such as Ca^2+^/H^+^ exchangers, or a concerted activity of multiple exchangers as proposed for secretory organelles and LROs which exploit the acidic pH of these organelles for Ca^2+^ import [51, 53, 59, 92, 93]. Recently TMEM165 was found to be involved in Ca^2+^ uptake of lysosomes [94, 95]. Interestingly, the ATP2C1 Ca^2+^ pump was found enriched in the WPB-associated proteome [85] although we could not confirm a colocalization with VWF by conventional immunofluorescence or ectopic expression of a HA-tagged construct. However, siRNA mediated depletion of ATP2C1 in HUVEC resulted in a minor but significant reduction of Ca^2+^ levels in WPB. This suggests that ATP2C1 present on WPB or the TGN is in part responsible for elevated Ca^2+^ in WPB.

Instead of being taken up from the cytosol, Ca^2+^ can also be transported directly between two organelles via membrane contact sites (MCS). Such Ca^2+^ exchange has been described for ER-mitochondria, ER-TGN or ER-lysosome MCS [96–100]. In a recent study, contacts between mitochondria and WPB were identified supporting the idea that other Ca^2+^ stores could also be involved in generating and maintaining elevated Ca^2+^ concentrations in WPB [101].

In summary, we introduce here an approach to visualize intra-organelle Ca^2+^ levels by combining specific Halo-tag targeting with the use of Ca^2+^ binding fluorescent dyes permeable to cells. This approach enabled us to show that endothelial WPB harbor elevated Ca^2+^ levels most likely required to support proper tubulation and packing of VWF tubules which is a prerequisite for optimal unfurling of VWF upon secretion into the blood vessel.

## Material and methods

### Cell culture and transfection

HUVEC were acquired from PromoCell as cryoconserved pools (C-12203) and cultured on Corning CellBind dishes at 37 °C and 5% CO_2_ in 1:1 mixed medium comprising M199 medium (PAN-Biotech) supplemented with 10% FCS, 30 µg/ml gentamycin, 0.015 µg/ml amphotericin B, and ECGMII (PromoCell) supplemented with 30 µg/ml gentamycin, 0.015 µg/ml amphotericin B. Experiments were conducted with HUVEC passage 3–5.

HUVEC were transfected using the Amaxa nucleofection system (Lonza) according to the manufacturer´s specifications. Per cuvette, cells from a confluent 20 cm^2^ dish together with 1–7 µg plasmid DNA and/or 400 pmol siRNA were resuspended in transfection buffer (4 mM KCl, 10 mM MgCl_2_, 10 mM sodium succinate, 100 mM NaH_2_PO_4_, pH 7.4 adjusted with NaOH). For depletion of proteins using siRNA, HUVEC were transfected with 400 pmol of siRNA twice. 48 h after the first transfection the cells were transfected again with the same amount of the respective siRNA. 24 h after the second transfection cells were subjected to live cell microscopy or lysed for Western blot analysis.

For live cell microscopy, transfected HUVEC were seeded on 8-chamber µ-slides (Ibidi, 80827) which were freshly coated with collagen from rat tail (Advanced Biomatrix, 5056) at a concentration of 50 µg/ml in 0.02 M acetic acid solution. Right before imaging HUVEC, the mixed medium was exchanged with Hank´s Balanced Salt Solution (Sigma, H6648), herein referred to as HBSS, supplemented with 20 mM HEPES pH 7.0–7.6 (Sigma, H0887), 1 mM MgCl_2_ and 0.9 mM CaCl_2_.

### Plasmids and siRNA

For generation of P-sel-lum-GCaMP6s-mApple the soluble luminal domain of P-selectin, herein referred to as P-sel-lum, was amplified from P-sel-lum-mRFP [44] using the following primers: Pr. Fw 5´-ATGGCGAACTGCCAAATAGCCATCTTGTACC, Pr. Rev 5´-CGGCTTCCTGGATAGTCAATGGTCCT and inserted into a GCaMP6s-mApple backbone by using the NEBuilder Hifi DNA Assembly. For the amplification of the GCaMP6s-mApple backbone from TPCN2-GCaMP6s-mApple [91] the following primers were used: Pr. Fw 5´-ATTGACTATCCAGGAAGCCGGCATGACTGGTGGACAGC, Pr. Rev 5´-GCTATTTGGCAGTTCGCCATTGAATTCGAAGCTTGAGCTC.

VWF-mRFP-Halo was obtained by amplification of the HaloTag sequence from pHaloTag-EGFP (Addgene Plasmid No: 86629) and its insertion into a VWF-mRFP plasmid, which was kindly provided by Tom Carter (St. George´s University of London, UK) [102, 103] using NEBuilder Hifi DNA Assembly. The following primers were used: Pr. Fw Halo 5´-TCCACCGGCGCCCTGTACAAAGCCACCATGGCAGAAATC, Pr. Rev Halo 5´-AGGTTCAGGGGGAGGTGTGGTTAGCCGGAAATCTCGAGCGT, Pr. Fw VWF-mRFP backbone 5´-CCACACCTCCCCCTGAACCTG, Pr. Rev VWF-mRFP backbone 5´-TTGTACAGGGCGCCGGTG.

mRFP-Halo-LAMP1 was obtained by exchanging RpHLuorin2 in LAMP1-RpHLuorin2 (Addgene #171720) with mRFP-Halo amplified from VWF-mRFP-Halo using NEBuilder Hifi DNA Assembly. For the amplification of the respective fragments the following primers were used: Pr. Fw mRFP-Halo 5´-CCGGTCATGGCCTCCTCC, Pr. Rev mRFP-Halo 5´-GCCGGAAATCTCGAGCGTCG, Pr. Fw LAMP1 backbone 5´- CGACGCTCGAGATTTCCGGCGGCTCAGGCTCAGCAATGTTTATGGTG, Pr. Rev LAMP1 backbone 5´-TCGGAGGAGGCCATGACCGGGGTGGCGACCGGTGTTGC.

Halo-mRFP was obtained by digestion of pHaloTag-EGFP (Addgene Plasmid No: 86629) and the pmRFP1-N1 vector from Addgene (Plasmid No: 54635) with NheI/KpnI restriction enzymes and the subsequent insertion of the HaloTag sequence in the multiple cloning site of the plasmid.

ATP2C1-HA was kindly provided by Julia von Blume.

siRNA targeting ATP2C1 was obtained from Horizon Discovery (L-006119-00-0010). AllStars Negative Control siRNA was from Qiagen (102781).

### Antibodies

For immunofluorescence and western blot analysis the primary and secondary antibodies listed in Table 1 were used.

### Western blot analysis

For preparation of cell lysates, HUVEC were harvested using trypsin/EDTA. After washing once with PBS, cell pellets were resuspended in 30 µl RIPA buffer (25 mM Tris-HCl, 150 mM NaCl, 0.1% (w/v) SDS, 0.5% (w/v), 1% (v/v) Triton X-100, pH 7.5) supplemented with 1x Complete protease inhibitor cocktail (Roche, 04693116001) per 20 cm^2^ confluent HUVEC dish and lysed for 15 min on ice. Cellular debris was removed by centrifugation at 1250 x g for 10 min at 4°C. Protein loading buffer was added to a final concentration of 1x and the samples were incubated for 10 min at 95°C.

Samples were subjected to 10% SDS-PAGE for 30 min at 70 V and subsequently at 120 V, and blotted onto 0.2 µm nitrocellulose membrane in a wet tank system at 115 V for 1h at 4°C in Tris-Glycine buffer (25 mM Tris, 190 mM glycine, 20% (v/v) methanol). Membranes were blocked using 3% skim milk in TBST (150 mM NaCl, 20 mM Tris-HCl, 0.1% (v/v) Tween-20, pH 7.4) for at least 30 min and incubated with primary antibodies (see Table 1) over night at 4°C. For signal detection, infrared conjugated secondary antibodies (IRdye680RD or IRdye800CW, LICOR) and the Odyssey imaging system (LICOR) were used.

### Experiments with HUVEC expressing P-sel-lum-GCaMP6s-mApple

Bafilomycin A1 (BafA1) was purchased from Cayman Chemical (Cay11038-1) and dissolved in DMSO. 24 h after seeding on 8-chamber μ-slides, HUVEC transfected with the P-sel-lum-GCaMP6s-mApple construct were incubated with media containing 250 nM BafA1 for 2 h or 0.1% DMSO for 3 h. Subsequently medium was exchanged with HBSS, supplemented with 20 mM HEPES pH 7.0–7.6, 1 mM MgCl_2_, 0.9 mM CaCl_2_ and 250 mM BafA1 or 0.1% DMSO, respectively, and cells were subjected to live cell imaging at 37˚C for less than 1 h.

### Loading of HUVEC with MaPCa-656

MaPCa-656_high_ and MaPCa-656_low_ have been described before [63]. The dyes were dissolved in DMSO to a final concentration of 2 mM and aliquots were stored at -20°C. 4 h or 24 h after seeding on 8-chamber µ-slides, HUVEC transfected with one of the mRFP-Halo constructs were incubated with freshly prepared media containing 1 µM MaPCa-656_high/low_ or 0.05% DMSO for 20 h or 2 h at 37 °C. Subsequently medium was exchanged with HBSS, supplemented with 20 mM HEPES pH 7.0–7.6, 1 mM MgCl_2_, 0.9 mM CaCl_2_ and 1 µM MaPCa-656_high/low_ or 0.05% DMSO, respectively, and cells were subjected to live cell imaging at 37 °C for less than 1 h. Solutions containing MaPCa-656_high/low_ were freshly prepared.

### Acceptor photobleaching

HUVEC expressing VWF-mRFP-Halo were treated with 1 µM MaPCa-656_high_ for 2 h as described above and were subsequently subjected to acceptor photobleaching (APB) during live cell microscopy. APB was performed with an LSM 780 microscope (Carl Zeiss) equipped with a Plan-Apochromat 63×/1.4 oil immersion objective. mRFP and MaPCa-656_high_ fluorescence of a single WPB was recorded in a single plane for 5 frames prior to bleaching of MaPCa-656_high_ using a 633 nm laser. Thereafter acquisition was continued for 15 frames. mRFP and MaPCa-656_high_ fluorescence was normalized to the respective intensity value before bleaching. FRET efficiency was calculated according to the following equation:

$$E(\%)=\frac{{average\;normalized\;I}_{RFP\;(post-bleach)}-{average\;normalized\;I}_{RFP\;(pre-bleach)}}{{average\;normalized\;I}_{RFP\;(post-bleach)}}\times 100$$

### Immunofluorescence staining

Cells were cultivated on collagen coated coverslips (12 mm diameter) until they reached confluency, then fixed in 4% PFA in PBS for 10 min at RT and permeabilized using 0.1% Triton X-100 in PBS for 2 min. Unspecific binding was blocked by addition of 3% BSA in PBS for at least 30 min, followed by antibody incubation (see Table 1) over night at 4°C in 3% BSA in PBS. Secondary antibodies (see Table 1) were diluted 1:400 and incubated for 45 min at room temperature (RT). After extensive washing, samples were mounted in mounting medium.

### Microscopy

Confocal microscopy of fixed cells was performed using an LSM 800 or LSM 980 microscope (Carl Zeiss) equipped with a Plan-Apochromat 63×/1.4 oil immersion objective. Live cell imaging was conducted with a LSM 780 microscope (Carl Zeiss) using a Plan-Apochromat 63×/1.4 oil immersion objective and a microscope stage maintained at 37 °C.

For live cell imaging of HUVEC expressing P-sel-lum-GCaMP6s-mApple multi-channel acquisition was achieved using 2 tracks for GCaMP6s and mApple on a spectral GaAsP-PMT [GCaMP6s (excitation laser 488, detection wavelengths: 490–540 nm), mApple (excitation laser 561, detection wavelengths: 450–700 nm)].

For live cell imaging involving MaPCa-656 indicators multi-channel acquisition was achieved using 2 tracks for mRFP and MaPCa-656 on a spectral GaAsP-PMT [mRFP (excitation laser 561, detection wavelengths: 560–615 nm), MaPCa-656 (excitation laser 633, detection wavelengths: 640–694 nm)]. Z-stacks (7 planes with 0.410 µm spacing) were acquired with 0.090 µm pixel size, 1.23 µs pixel integration time and 2x averaging at a bit depth of 8-bit.

### Image analysis

Confocal images were analyzed using Fiji [104]. For measurements of Feret diameter or fluorescence intensities, WPB were identified based on the mRFP signal of maximum intensity projections. Segmentation of WPB was achieved by training a pixel classification model using Ilastik [105]. The images were analyzed using a custom macro in Fiji. Maximum intensity projections of the multicolor images were created. For WPB segmentation, the pixel classification model was applied through the Ilastik plugin for Fiji. For further segmentation, the watershed plugin of Fiji was used. Identified particles smaller than 0.11 µm^2^ were excluded from quantification. For each identified particle the Feret diameter and the mean gray value for mRFP and MaPCa were determined from the gray values of all its associated pixels in the segmented area. Calcium levels were assessed by normalizing MaPCa signal of WPB to the expression level of the construct, i.e. the mRFP signal.

To quantify the normalized MaPCa signal of WPB in relation to the nuclear distance, Zeiss Arivis Pro (version 4.1) was used. The analysis was conducted with maximum intensity projections of z-stacks. Nuclei were segmented manually by drawing an outline around the negative staining in the nuclear region. WPB were segmented using a machine learning classifier followed by watershed splitting. Identified particles smaller than 0.11 µm^2^ were excluded from quantification. All distances from WPB to the nuclear perimeter were measured and categorized in perinuclear (<7 µm distance to the nucleus perimeter) and peripheral (≥7 µm distance).

### Statistics

All statistics were performed using GraphPad PRISM (10.1.0). Asterisks mark statistically significant results: ****p ≤ 0.0001, ***p ≤ 0.001, **p ≤ 0.01, *p ≤ 0.05. Normal distribution was assessed by the Shapiro-Wilk or the D´Agostino & Pearson test. Normally distributed data was analyzed employing an unpaired t-test with Welch´s correction. Non-parametric data was analyzed using a Mann-Whitney test.

## Supporting information

S1 File Supporting information file containing multiple supporting figures. (DOCX)
