# Supplementary material for: Chemigenetic Ca2+ indicators report elevated Ca2+ levels in endothelial Weibel-Palade bodies
Source: PLoS One. 2025 Jan 27;20(1):e0316854. doi: 10.1371/journal.pone.0316854 (PMC11771901; doi:10.1371/journal.pone.0316854)
Supplement: S1 File — (DOCX) [file pone.0316854.s001.docx]

**Supporting Information file containing multiple supporting figures**

**Suppl. Fig. S1**

**
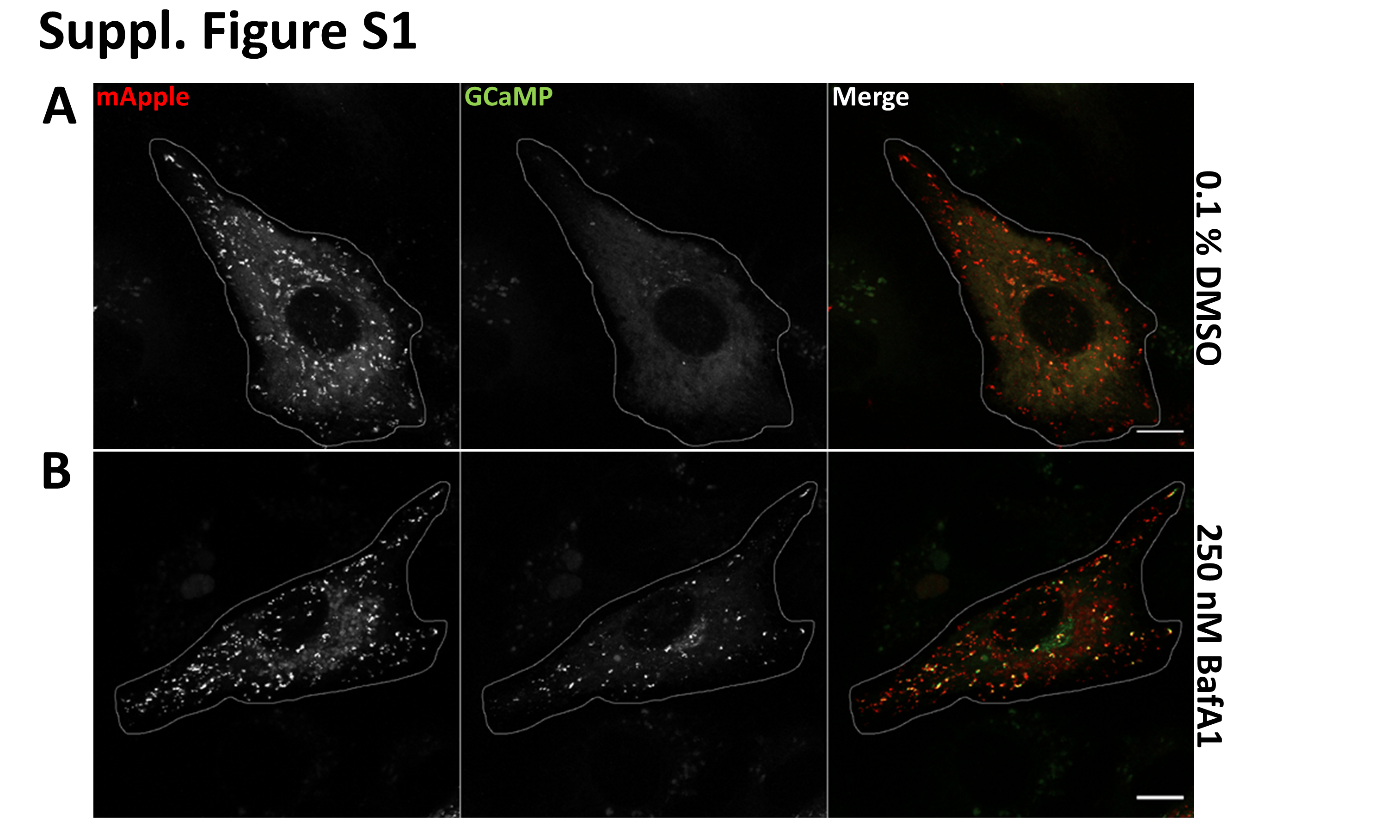
**

**Supplemental Figure S1. Fluorescence of the genetically encoded Ca^2+^ indicator GCaMP is quenched in the acidic milieu of WPB.** 24 h post transfection, HUVEC expressing GCaMP6s and mApple both fused in tandem to the WPB localizing P-sel-lum were incubated with 0.1% DMSO (**A**) or 250 nM of the V-ATPase inhibitor BafA1 (**B**), respectively, at 37 °C for 2 h and subsequently subjected to live cell imaging. Shown are images of maximum intensity projections of z-stacks. Cell circumferences are indicated by lines. Note that the GCaMP6s fluorescence is quenched in acidic WPB, i.e. no signal is visible in the mApple labeled WPB (DMSO treatment), whereas neutralization achieved by inhibition of the V-ATPase proton pump with BafA1 results in a clear GCaMP6s signal in WPB. Scale bars: 10 μm.

**Suppl. Fig. S2**


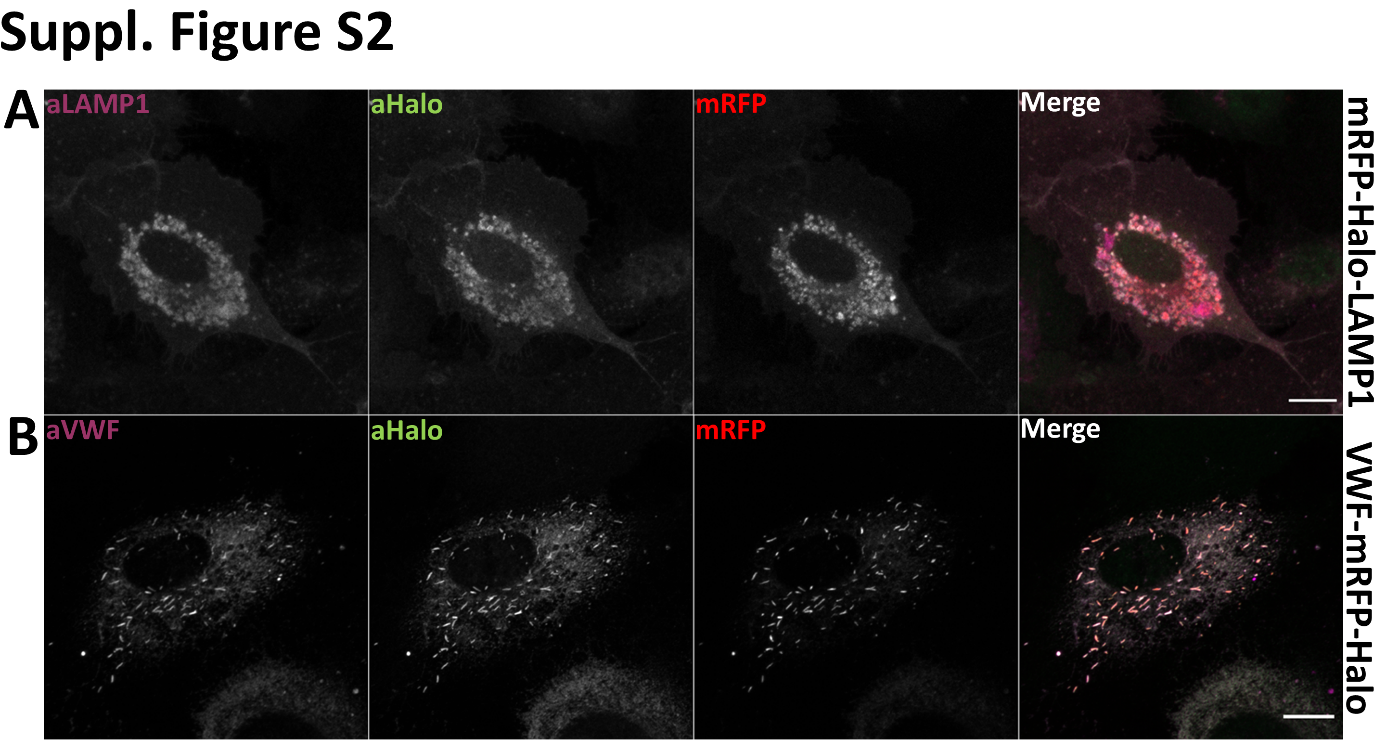


**Supplemental Figure S2. mRFP-Halo can be targeted to lysosomes and WPB in HUVEC.** 24 h post transfection with plasmids encoding mRFP-Halo-LAMP1 (**A**) or VWF-mRFP-Halo (**B**), respectively, confluent HUVEC were fixed and stained for LAMP1 as a general lysosome marker or VWF as a WPB protein (magenta) and for HaloTag (green) using the respective antibodies. Shown are confocal microscopy images displaying maximum intensity projections of z-stacks. Note that LAMP1 and VWF served successfully as organelle targeting sequences for the mRFP-Halo moiety. Scale bar: 10 μm.

**Suppl. Fig. S3**

**
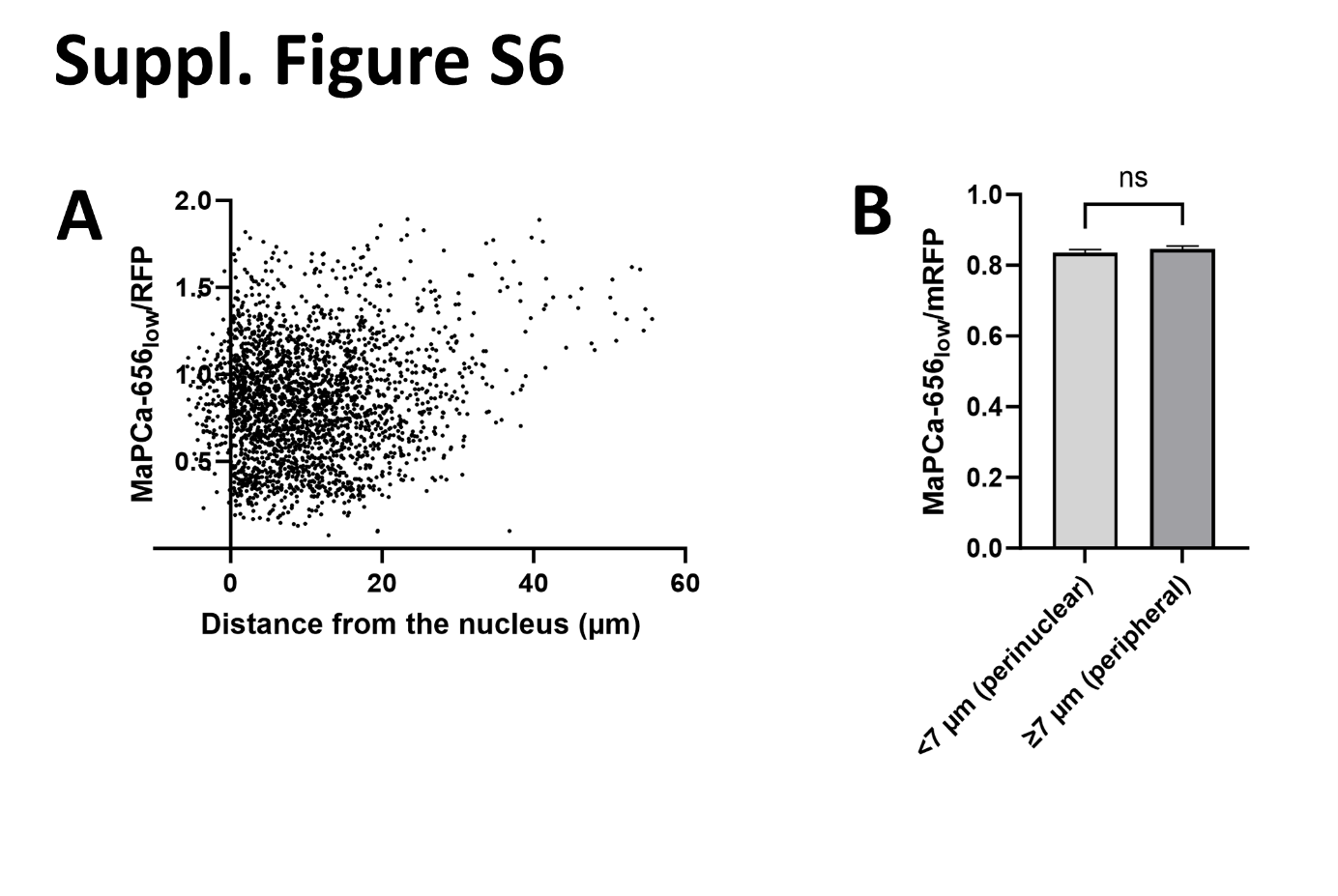
**

**Supplemental Figure S3. Intraluminal Ca^2+^ of WPB as a function of distance from the nucleus.** 24 h post transfection HUVEC, expressing VWF-mRFP-Halo were incubated with 1 µM MaPCa-656_low_ for 2 h at 37°C and subsequently subjected to live cell imaging. **A.** For each segmented WPB the intensity of MaPCa-656_low_ in relation to mRFP was measured and its distance to the nuclear envelope/perimeter was determined. In total 2534 WPB were measured in 3 independent experiments. **B.** WPB within a distance <7 µm from the nuclear envelope/perimeter were defined as perinuclear and WPB with a distance ≥7 µm were defined as peripheral. Bars indicate the mean. Error bars show SEM. Statistics were conducted using a Mann-Whitney test. ****p≤ 0.0001, ***p≤ 0.001, **p≤0.01, *p≤0.05, ns p>0.05.

**Suppl. Fig. S4**


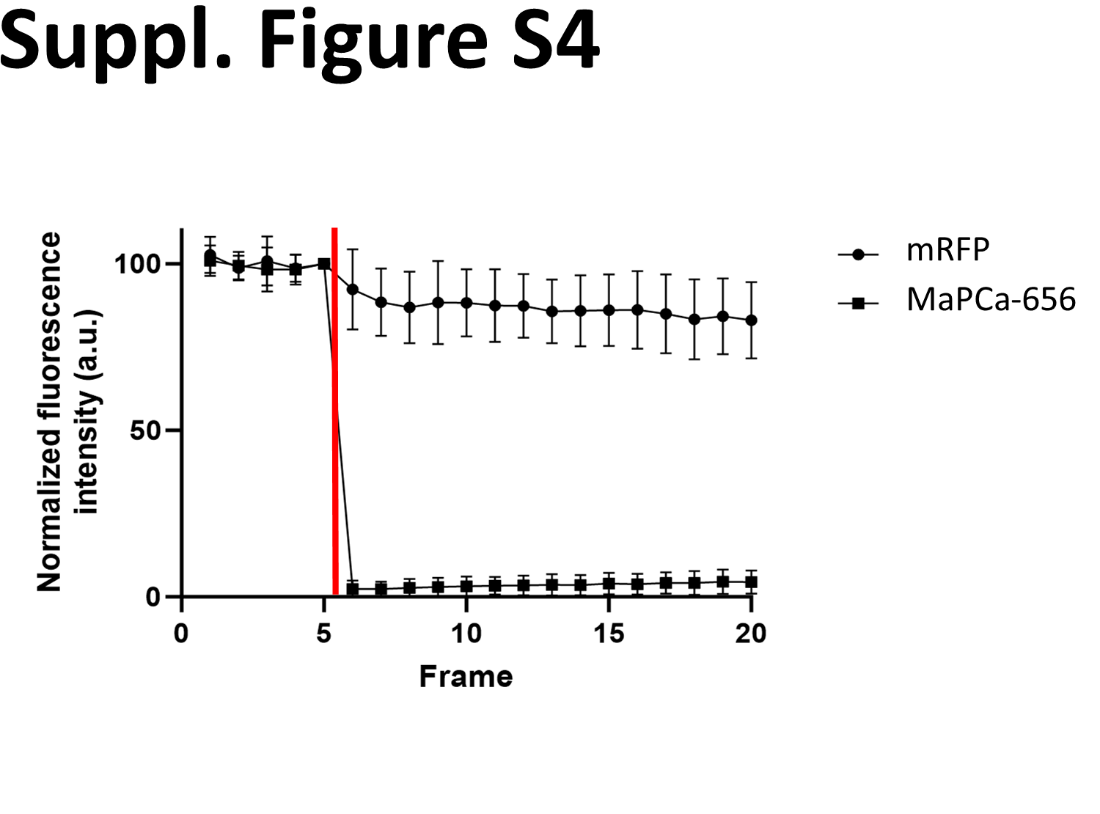


**Supplemental Figure S4. Acceptor photobleaching analysis of VWF-mRFP-Halo positive WPB loaded with MaPCa-656_high_.** 24h after transfection HUVEC were incubated with 1 µM MaPCa-656_high_ for 2 h at 37 °C and subjected to live cell microscopy. mRFP and MaPCa-656_high_ intensity were recorded over time. Bleaching of the potential FRET acceptor MaPCa-656_high_ was conducted after 5 frames as indicated by the red line. Data is normalized to the intensity value before bleaching. Shown is the mean with SD. n = 14.

**Suppl. Fig. S5**


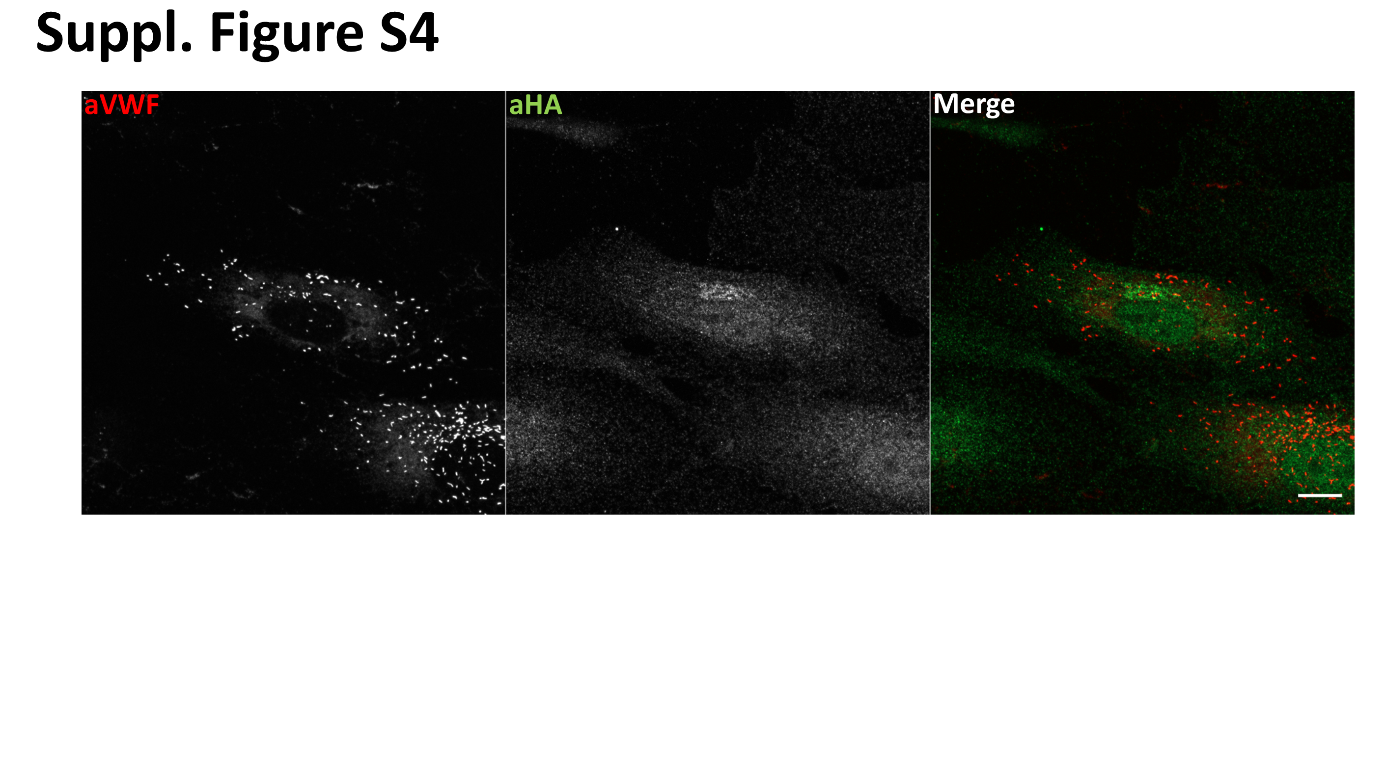


**Supplemental Figure S5. Localization of ectopically expressed ATP2C1-HA in HUVEC.** 24 h post transfection with an ATP2C1-HA construct, confluent HUVEC were fixed and stained for VWF as a general WPB marker (red) and for the HA-tagged ATP2C1 (green) using the respective antibodies. Shown are confocal microscopy images displaying maximum intensity projections of z-stacks. Scale bar: 10 μm.

**Suppl. Fig. S6**


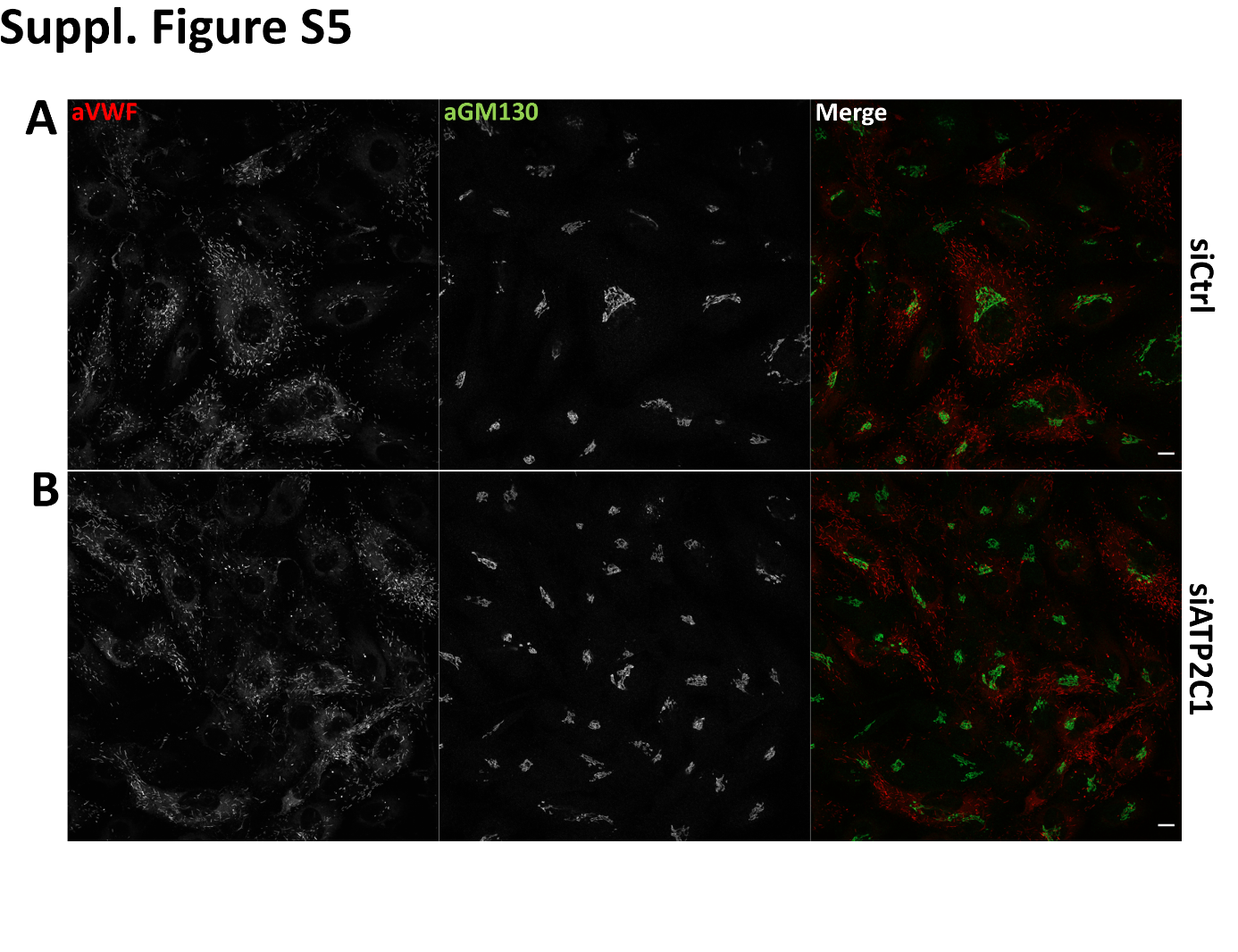


**Supplemental Figure S6. siRNA-mediated knockdown of ATP2C1 in HUVEC.** 24 h post transfection with siCtrl (**A**) and siATP2C1 (**B**), confluent HUVEC were fixed and stained for VWF as a WPB marker (red) and for GM130 as a Golgi marker (green) using the respective antibodies. Shown are confocal microscopy images displaying maximum intensity projections of z-stacks. Scale bar: 10 μm.

**Suppl. Fig. S7**

**
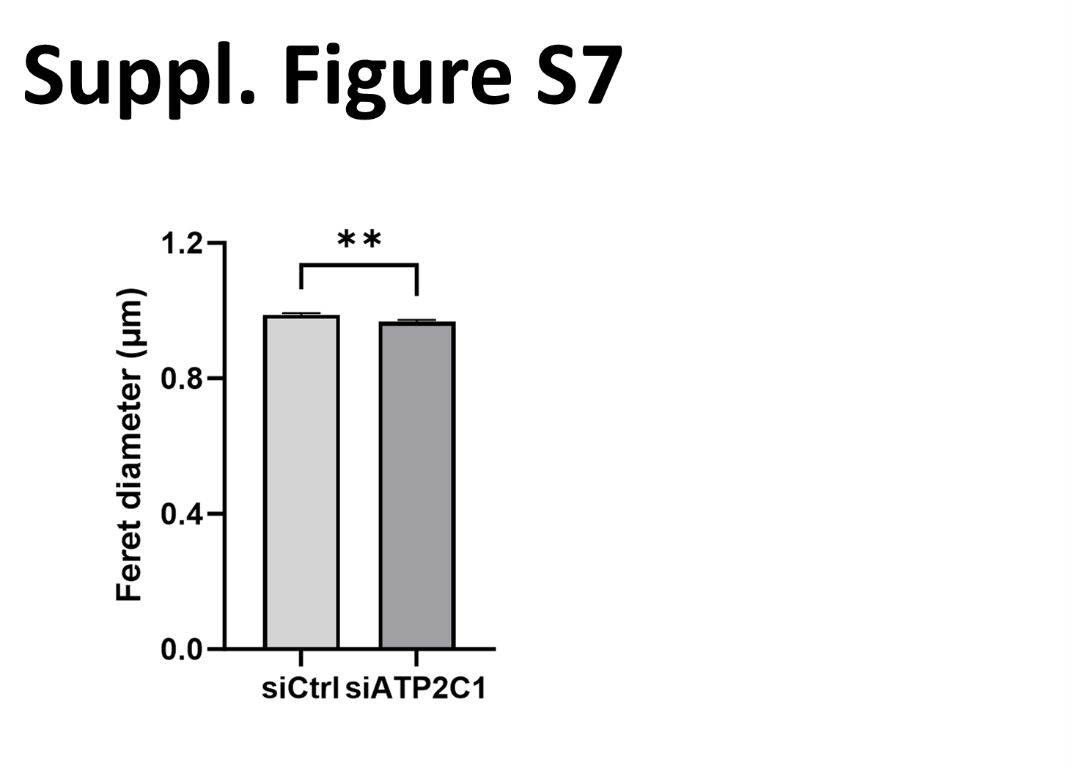
**

**Supplemental Figure S7. Knockdown of ATP2C1 has a minor impact on WPB size.** HUVEC transfected with the respective siRNA were additionally transfected with VWF-mRFP-Halo and incubated with 1 µM MaPCa-656_low_ for 2 h at 37 °C. MaPCa-656 and mRFP intensities of single WPB were measured during live cell microscopy in 3 experiments (siCtrl: 5943 WPB, siATP2C1: 6285 WPB). Bars indicate the mean. Error bars show SEM. Statistics were conducted using a Mann-Whitney test. ****p≤ 0.0001, ***p≤ 0.001, **p≤0.01, *p≤0.05.

**Suppl. Fig. S8**


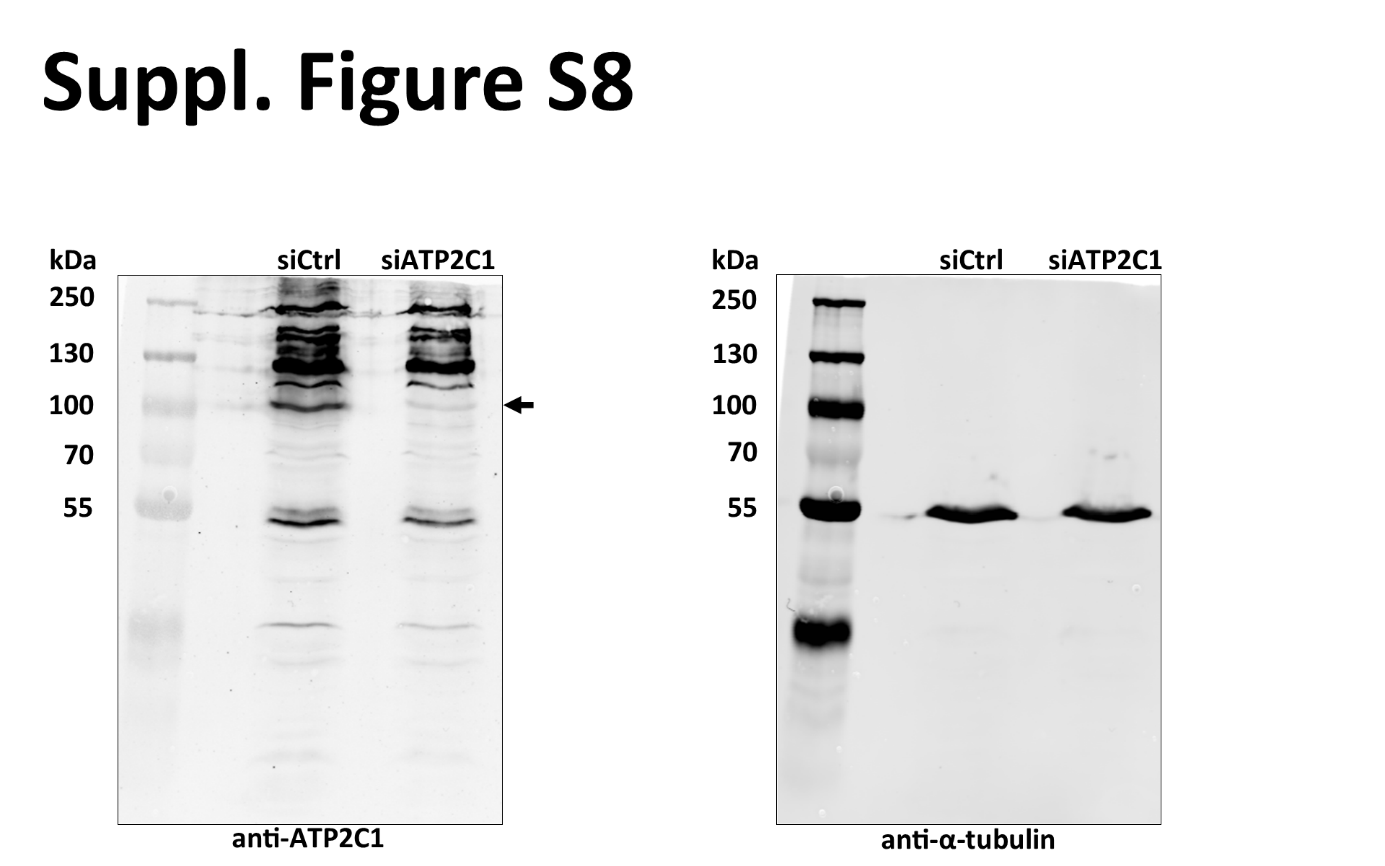


**Supplemental Figure S8.** **Knockdown of ATP2C1.** HUVEC were lysed 24 h after transfection with the respective siRNA and lysates were subjected to SDS-PAGE followed by Western blotting with anti-ATP2C1 or anti-α-tubulin antibodies. Arrow indicates the position of the ATP2C1 signal.
